# Novel HSAN1 Mutation in Serine Palmitoyltransferase Resides at a Putative Phosphorylation Site That Is Involved in Regulating Substrate Specificity

**DOI:** 10.1007/s12017-014-8339-1

**Published:** 2015-01-08

**Authors:** Daniela Ernst, Sinéad M. Murphy, Karthik Sathiyanadan, Yu Wei, Alaa Othman, Matilde Laurá, Yo-Tsen Liu, Anke Penno, Julian Blake, Michael Donaghy, Henry Houlden, Mary M. Reilly, Thorsten Hornemann

**Affiliations:** 1 Institute for Clinical Chemistry, University Hospital Zurich, CH-8091 Zurich, Switzerland; 2 Institute of Physiology and Zurich Center for Integrative Human Physiology (ZIHP), University of Zurich, CH-8057 Zurich, Switzerland; 3 Department of Neurology, Adelaide & Meath Hospitals Incorporating the National Children’s Hospital, Tallaght, Dublin 24, Ireland; 4 Academic Unit of Neurology, Trinity College Dublin, Dublin, Ireland; 5 Department of Clinical Neurophysiology, The National Hospital for Neurology and Neurosurgery, London, UK; 6 Department of Molecular Neuroscience, UCL Institute of Neurology, London, UK; 7 Department of Clinical Neurophysiology, Norfolk and Norwich University Hospital, Norwich, UK; 8 Nuffield Department of Clinical Neuroscience, University of Oxford, John Radcliffe Hospital, Oxford, UK; 9 Theodor Kocher Institute, University of Bern, Bern, Switzerland; 10 Life and Medical Sciences (LIMES) Institute, University of Bonn, Bonn, Germany; 11 Competence Center for Systems Physiology and Metabolic Diseases, Zuerich, Switzerland; 12 Department of Neurology, Neurological Institute, Taipei Veterans General Hospital, Taipei, Taiwan; 13 National Yang-Ming University School of Medicine, Taipei, Taiwan; 14 Department of Molecular Neurosciences, MRC Centre for Neuromuscular Diseases, UCL Institute of Neurology, Queen Square, London, UK

**Keywords:** Serine palmitoyltransferase, 1-Deoxysphingolipids, Sensory neuropathy, HSAN1, Sphingolipids

## Abstract

1-Deoxysphingolipids (1-deoxySL) are atypical sphingolipids that are formed by the enzyme serine palmitoyltransferase (SPT) due to a promiscuous use of l-alanine over its canonical substrate l-serine. Several mutations in SPT are associated with the hereditary sensory and autonomic neuropathy type I (HSAN1). The current hypothesis is that these mutations induce a permanent shift in the affinity from l-serine toward l-alanine which results in a pathologically increased 1-deoxySL formation in HSAN1 patients. Also, wild-type SPT forms 1-deoxySL under certain conditions, and elevated levels were found in individuals with the metabolic syndrome and diabetes. However, the molecular mechanisms which control the substrate shift of the wild-type enzyme are not understood. Here, we report a novel SPTLC2–S384F variant in two unrelated HSAN1 families. Affected patients showed elevated plasma 1-deoxySL levels and expression of the S384F mutant in HEK293 cells increased 1-deoxySL formation. Previously, S384 has been reported as one of the two (S384 and Y387) putative phosphorylation sites in SPTLC2. The phosphorylation of wild-type SPTLC2 was confirmed by isoelectric focusing. The impact of an S384 phosphorylation on SPT activity was tested by creating mutants mimicking either a constitutively phosphorylated (S384D, S384E) or non-phosphorylated (S384A, Y387F, Y387F+S384A) protein. The S384D but not the S384E variant was associated with increased 1-deoxySL formation. The other mutations had no influence on activity and substrate affinity. In summary, our data show that S384F is a novel mutation in HSAN1 and that the substrate specificity of wild-type SPT might by dynamically regulated by a phosphorylation at this position.

**Electronic supplementary material:**

The online version of this article (doi:10.1007/s12017-014-8339-1) contains supplementary material, which is available to authorized users.

## Introduction

Hereditary sensory and autonomic neuropathy type I (HSAN1) is an autosomal dominant ulceromutilating neuropathy with variable motor involvement caused by mutations in six genes: *SPTLC1* (Bejaoui et al. 2001; Dawkins et al. 2001), *SPTLC2* (Rotthier et al. 2010), *ATL1* (Guelly et al. 2011), *RAB7* (Verhoeven et al. 2003), *DNMT1* (Klein et al. 2011) and *ATL3* (Kornak et al. 2014).

*SPTLC1* and *SPTLC2* encode for two of three subunits of the enzyme serine palmitoyltransferase (SPT) which catalyzes the condensation of palmitoyl-CoA with l-serine—the first and rate-limiting step in ceramide de novo synthesis (Hanada 2003). SPT is catalytically promiscuous and can also metabolize l-alanine and glycine under certain conditions (Supplementary Figure 1). This alternative activity forms an atypical class of neurotoxic 1-deoxy-sphingolipids (1-deoxySL) which lack the C1 hydroxyl group of regular sphingolipids (Zitomer et al. 2009; Penno et al. 2010). They are not able to form higher substituted sphingolipids like sphingomyelins or glycosphingolipids and can also not degrade via the canonical pathway which requires the formation of S1P as a catabolic intermediate. The HSAN1 mutations in SPT induce a permanent shift from l-serine toward l-alanine which results in a pathologically increased 1-deoxySL formation (Penno et al. 2010; Rotthier et al. 2010). Elevated 1-deoxySL levels were found in plasma and lymphoblasts of HSAN1 patients and in plasma and peripheral nerve tissue of transgenic mice (Penno et al. 2010; Rotthier et al. 2010; Garofalo et al. 2011). 1-deoxySL are neurotoxic and impair neurite formation in cultured neurons (Penno et al. 2010).

Also, wild-type SPT can metabolize l-alanine under certain conditions. Elevated 1-deoxySL levels were found in individuals with the metabolic syndrome and type 2 diabetes and might also be involved in the pathology of diabetic neuropathy (Othman et al. 2014). However, the molecular mechanism which controls the substrate shift of the wild-type enzyme is not understood. Here, we report a novel HSAN1 mutation in *SPTLC2* (S384F) which was identified in two unrelated families. The mutation is associated with significantly elevated plasma 1-deoxySL levels. The same residue (S384) was reported previously as a putative SPTLC2 phosphorylation site (Olsen et al. 2006). We therefore investigated whether a phosphorylation of S384 has an influence on substrate specificity of SPT and whether this regulation is impaired in the two newly identified HSAN1 families.

## Materials and Methods

### Ethical Approval

Ethical approval for this study was obtained from the Joint Medical and Ethics Committee at the National Hospital for Neurology and Neurosurgery (NHNN). Written informed consent was obtained from all patients.

### Patients

One hundred and seven patients with HSAN were selected from our inherited neuropathy database. The database includes patients seen in the peripheral neuropathy clinics in the NHNN, as well as patients whose DNA was referred from other hospitals for diagnostic and research testing. All patients selected had a clinical diagnosis of HSAN, presenting with progressive distal sensory loss, with or without ulceromutilating complications or autonomic dysfunction. Because of the overlap between Charcot-Marie-Tooth Type 2B (CMT2B) and HSAN1, patients with motor involvement were included; however, sensory features were always predominant. Diagnosis was based on clinical phenotype in addition to neurophysiology. All patients were negative for mutations in *SPTLC1* and most were also negative for mutations in *RAB7*, *NGFB*, *FAM134B* and *NTRK1*. Four hundred and seventy-eight British control chromosomes were screened for the S384F mutation.

### Patient Assessment

All patients found to have mutations were seen and had detailed clinical and neurophysiological assessments performed, including assessment of neuropathy severity using the Charcot-Marie-Tooth Neuropathy Score 2 (CMTNS2) (Murphy et al. 2011). Nerve conduction studies were performed using standard techniques.

### Genetic Sequencing

All 12 exons and flanking introns of *SPTLC2* were amplified using Roche polymerase chain reaction (PCR) reagents. Sequence reactions were performed using Big Dye Terminator version 3.1 Cycle Sequencing Kit (Applied Biosystems) and resolved on an ABI 3730xl Sequencer. Sequence variants were confirmed by repeat sequencing. Three commonly used prediction programmes, PolyPhen2 (http://genetics.bwh.harvard.edu/pph/), SIFT (http://blocks.fhcrc.org/sift/SIFT.html) and aGVGD (http://agvgd.iarc.fr/), were used to predict the effect of the mutations on protein function.

**Table:** 

SPTLC2 exon	Forward primer	Reverse primer	Primer conditions
1	CCTACAGAGCCTGCCTTG	CGGTGTGGACTGGCGGAG	58 touchdown
2	GGTATAATTCAGCAAATCTC	TTTAACTGCATCTGGAATAG	60–50 touchdown
3	TAATGAAATTGCCCTTATAC	AATCATATTGTATCCTCAGC	60–50 touchdown
4	ATAGACTTTGTTCTCTCTGC	CTAAATGACATGACAAAGTG	60–50 touchdown
5	TCTGAAAAGGACACAACAC	TTTAGCTCACTCTGACTGC	51.3
6	AGCTATTAGTGTTTGTGGC	TCATTTATACTTTCAAGTGC	60–50 touchdown
7	TATCTGAGGCATGGTTTC	TAGACTAATGTTCCCTTCAG	65–55 touchdown
8	ATAATAATGAAGTGCCAAAC	GTATTATGAGCCTAAACCAG	60–50 touchdown
9	TCTAGAACTTAGAAGGAAAGG	TGCCTATTAGTAAACCTGAC	60–50 touchdown
10	GATAGAATGGAGATAGAGGAG	TAAGGACAAGACCATTTTC	60–50 touchdown
11	TTGAAATCTTTGAGGACAG	GCTCACAAGAACATCAAG	60–50 touchdown
12	GCACTAGACATAAGTCCTGC	ACAGAAGTGTGGTTCCTG	60–50 touchdown

PCR comprised the following steps for all exons except exons 1 and 5:

- (1) 95 °C for 15 min, (2) 25 cycles of 95 °C for 30 s, 60 °C (reduced by 0.4 °C per cycle) for 30 s, and 72 °C for 45 s, (3) 13 cycles of 95 °C for 30 s, 50 °C for 30 s, and 72 °C for 45 s and (4) 72 °C for 10 min.

For exon 1:

- (1) 95 °C for 15 min, (2) 38 cycles of 95 °C for 30 s, 58 °C (reduced by 0.4 °C per cycle) for 30 s, and 72 °C for 45 s and (3) 72 °C for 10 min. 10 % DMSO

For exon 5:

- (1) 95 °C for 15 min, (2) 37 cycles of 95 °C for 30 s, 51.3 °C (reduced by 0.4 °C per cycle) for 30 s, and 72 °C for 45 s and (3) 72 °C for 10 min.

### Cloning

*SPTLC2* cDNA was amplified by PCR from a cDNA library and cloned into a mammalian pcDNA 3.1D/V5-His-TOPO expression vector. The *SPTLC2* mutations S384D, S384E, S384A, S384F, S384A+Y387F, Y387F and G382V were introduced by site-directed mutagenesis (primer sequences below). All constructs were verified by sequencing.

**Table:** 

S384D_fw:	5′-TCA CAA AGA GTT TTG GTG CTG ATG GAG GAT ATA TTG GAG GC-3′
S384D_RV:	5′-GCC TCC AAT ATA TCC TCC ATC AGC ACC AAA ACT CTT TGT GA-3′
S384E_FW:	5′-TTC ACA AAG AGT TTT GGT GCT GAT GGA GGA TAT ATT GGA GGC AAG-3′
S384E_RV:	5′-CTT GCC TCC AAT ATA TCC TCC CTC AGC ACC AAA ACT CTT TGT GAA-3′
S384F_FW:	5′-CAA AGA GTT TTG GTG CTT TCG GAG GAT ATA TTG GAG-3′
S384F_RV:	5′-GCC TCC AAT ATA TCC TCC GAA AGC ACC AAA ACT CTT-3′
S384A_FW:	5′-CAC AAA GAG TTT TGG TGC TGC AGG AGG ATA TAT TGG AGG CA-3′
S384A_RV:	5′-TGC CTC CAA TAT ATC CTC CTG CAG CAC CAA AAC TCT TTG TG-3′
S384AY387F_FW:	5′-CAA AGA GTT TTG GTG CTG CTG GTG GAT TTA TTG GAG GCA AGA-3′
S384AY387F_RV:	5′-CCT TCT TGC CTC CAA TAA ATC CAC CAG CAG CAC CAA AAC TCT TTG-3′
Y387F_FW:	5′-GAG TTT TGG TGC TTC TGG TGG ATT TAT TGG AGG CAA GAA GG-3′
Y387F_RV:	5′-CCT TCT TGT CTC CAA TAA ATC CAC CAG AAG CAC CAA AAC-3′

### Stable Expression of Mutant and Wild-type SPTLC2 in HEK293 Cells

HEK293 cells (ATCC) were cultured in DMEM (Sigma-Aldrich) with 10 % fetal calf serum (FisherScientific FSA15-043) and penicillin/streptomycin (100 U/ml and 0.1 mg/ml, respectively, Sigma-Aldrich). Stable transfection was performed using TurboFect (Thermo Scientific), and cells were kept under selection with 400 μg/ml Geneticin (Gibco, Invitrogen). Expression of the constructs was confirmed by immune blotting and RT-PCR (Supplemental Figure 2 A, B).

### 2D-PAGE Analysis

Total proteins of CHO or Hek293 cells were extracted either in the presence or absence of a phosphatase-inhibitor cocktail (Roche). The aliquot without phosphatase inhibitor was treated with alkaline phosphatase (FastAP, Thermo Scientific) for 30 min at 37 °C. 1 mg of total protein each was mixed with DeStreak™ Rehydration Solution (Amersham Biosciences) to a final volume of 100 µl and loaded on a Immobiline™ DryStrip (GE Healthcare), together with 0.5 % IPG buffer (Amersham Biosciences) by passive rehydration over-night. Ampholites covered a pH range from pH 6–11. Iso-electric focusing was carried out on an Ettan IPGphor (Amersham Biosciences) according to following conditions: Step 1: 300 V, 200 kVh; step 2: 1,000 V, 300 kVh; step 3: 5,000 V, 4,500 kVh; step 4: 5,000 V, 2,000 kVh; and step 5: hold on 500 V. All steps were performed at 20 °C, for 3 h with a maximal current of 50 µA per strip. Focused proteins were subsequently equilibrated stepwise in 65 mM DTT and 135 mM iodoacetamide (both in 1 M Tris, 6 M Urea, 30 % v/v glycerol, 2 % w/v SDS, 0.01 % v/v bromphenolblue) with intermediate washing. Proteins were separated in the second dimension on a 12 % SDS-PAGE, followed by Western blotting (PVDF membrane) and immune detection using a polyclonal SPTLC2 antibody (Hornemann et al. 2006).

### Cell-Based SPT Activity Assay

A total of 200,000 HEK293 cells stably transfected with SPTLC2wt and SPTLC2 mutants (S384D, S384E, S384F, S384A, S384A+Y387F and Y387F) were seeded and cultured as described above. After 3 days, the conditioned medium was exchanged with l-serine and l-alanine deficient DMEM (Genaxxon BioScience). After a pre-incubation of two hours, isotope-labeled (2,3,3) d3 l-serine (1 mM) together with 5 mM (3C_13_)-labeled l-alanine (Cambridge Isotope Laboratories, Inc.) was added to the medium. After 24 h, the cells were washed twice with PBS, harvested and counted (Z2 Coulter Counter, Beckman Coulter). Cells were centrifuged (800×*g*, 5 min at 4 °C), and pellets were stored at −20 °C for lipid extraction and analysis. The isotope-labeled sphingoid bases are referred to as d2-SA/d2-SO (from d3 l-serine) or C^13^ doxSA/C^13^ doxSO (from 3C_13_ l-alanine).

### Kinetic Assay with l-Alanine

A total of 400,000 HEK293 cells stable expressing SPTLC2wt or SPTLC2–S384D were cultured as described above. At day two, 0–35 mM isotope-labeled l-alanine (Sigma) diluted in PBS was added to the cells without changing the media. Cells were cultured for 48 h, washed twice in PBS, harvested and counted (Z2 Coulter Counter, Beckman Coulter). Cells were centrifuged (800×*g*, 5 min at 4 °C), and pellets were stored at −20 °C until lipid extraction and acid–base extraction.

### SPT Activity Assay and Lipid Base Extraction

Total protein was extracted from frozen HEK293 cell pellets using SPT assay buffer (50 mM HEPES pH 8, 1 mM EDTA, 0.2 % Triton-X). 500 µg total protein was added to a mix of 160 µM palmitoyl-CoA (Sigma), 30 µM pyridoxal-5′-phosphate (Sigma) and either 64 mM l-serine or 320 mM l-alanine (Sigma). The mix was incubated for 45 min at 37 °C under constant shaking at 800 rpm (Thermomixer comfort, Eppendorf). The in vitro SPT reaction was stopped by adding 500 µl lipid extraction buffer containing 4 vol MetOH-KOH and 1 vol CHCl3 spiked with 200 pmol isotope-labeled d7-sphinganine and d7-sphingosine (Avanti Polar Lipids) as internal extraction standards. Lipids were base-extracted and analyzed on a TSQ Quantum Ultra MS analyzer (Thermo Scientific) as described earlier (Othman et al. 2012)

### Extraction and Analysis of Sphingoid Bases

500 microliters MetOH including 200 pmol internal standard (d7-sphinganine and d7-sphingosine, Avanti Polar Lipids) was added to 100 µl of plasma or PBS suspended cells. Lipid extraction was performed for 1 h at 37 °C with constant agitation at 1,000 rpm (Thermomixer comfort, Eppendorf). Precipitated protein was removed by centrifugation for 5 min at 16,000×*g,* and the supernatant was transferred to a new tube. Lipids were hydrolyzed by adding HCL (4.8 % v/v, for 16 h at 65 °C); after neutralization by adding KOH (1.5 M final concentration), the free sphingoid bases were extracted with chloroform (92.6 % v/v) under basic conditions (0.15 N ammoniac). The sphingoid base profile was analyzed on a TSQ Quantum Ultra MS analyzer (Thermo Scientific) as described earlier (Othman et al. 2012).

### Statistics

One-way ANOVA was used for statistical analysis. Significance was verified after Bonferroni multiple-correction. *p* values were stated according to the following definition: **** *p* < 0.0001, *** *p* < 0.001, ** *p* < 0.01 and * *p* < 0.05. Statistical analysis was performed using GraphPad Prism5.

## Results

### A Novel SPTLC2 Mutation S384F Was Found in Two Families

**Fig. 1:**
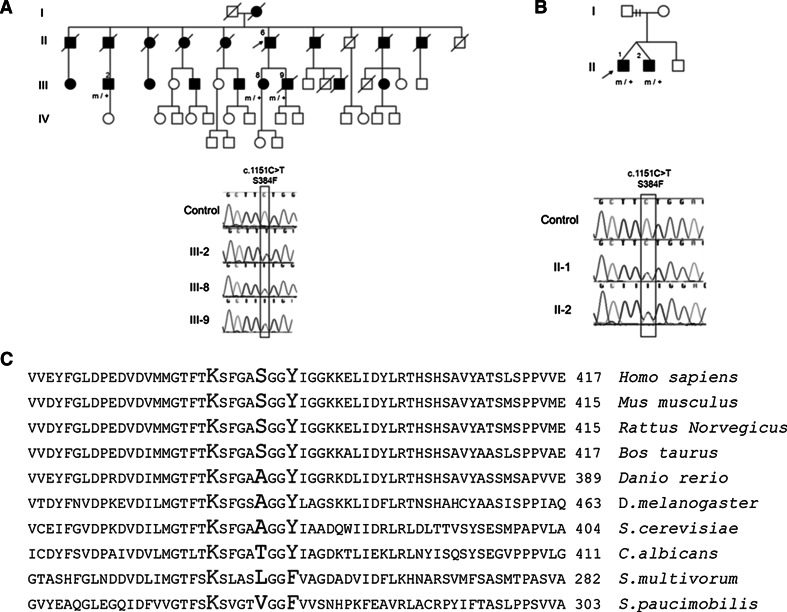
Pedigrees of the *SPTLC2*–S384F families and electropherogram of affected patients and control (**a**, **b**). *Square* = male; *circle* = female; *diagonal line* = deceased; *filled symbol* = affected; *m/+* = heterozygous for mutation; and *arrow* = proband. **c** Protein alignment of the SPTLC2 orthologous from human (*Homo sapiens*), mouse (*mus musculus*), rat (*Rattus norwegicus*), cattle (*Bos Taurus*), zebrafish (*Danio rerio*), fly (*Drosophila melanogaster*), baker’s yeast (*Saccharomyces cerevisiae*), *Candida albicans* and the two sphingolipid producing bacteria *Sphingomonas multivorum* and *Sphingomonas* *paucimobilis*

We identified a novel c.1151 C>T (S384F) *SPTLC2* variant in two unrelated HSAN1 index cases. This variant was not found in 478 control chromosomes nor annotated in the 1,000 genomes (http://www.1000genomes.org/) or the ESP6500 (http://evs.gs.washington.edu/EVS/) database. Further studies confirmed segregation of the mutation; the mutation was found in all affected family members who were tested (Fig. 1a, b). The S384 residue is conserved in mammals but replaced by alanine in lower vertebrates (Fig. 1c). PolyPhen2 and SIFT both predict that this change would be damaging.

### Clinical Details of S384F Patients

The two S384F families could not be genealogically linked (Fig. 1a). In comparison with other HSAN1 families, patients from the two S384F families had a rather late onset of disease in the fourth or fifth decade. Most patients presented with reduced sensation in the feet and later development of motor weakness. Sensory loss occurred in a glove and stocking distribution; pinprick was affected to a greater extent than vibration perception, similarly to patients with *SPTLC1* mutations (Houlden et al. 2006). Sensory complications including ulcers and accidental burns occurred in all patients, and some individuals had amputations. Motor involvement occurred in all families, though to a variable extent. The disease showed clinical heterogeneity even within families, e.g., Pedigree A (Fig. 1), III-8 having only mild distal lower limb weakness at 65 years, while her sibling III-9 had significant proximal and distal upper and lower limb weakness and required a wheelchair since 65 years. None of the patients had significant autonomic symptoms.

### Neurophysiology

Details of neurophysiology from the S384F patients are shown in Tables 1 and 2. Overall, nerve conduction studies demonstrated a sensorimotor axonal neuropathy. Sensory action potentials were absent in the lower limbs and reduced or absent in the upper limbs. Motor responses were absent or reduced in the lower limbs with normal or reduced amplitudes in the upper limbs. Upper limb motor conduction velocities were either normal or demonstrated some slowing, similar to what is seen in families with *SPTLC1* mutations (Houlden et al. 2006).

**Table 1:** Clinical details of *SPTLC2*–S384F patients

Family	Age last exam	Sex	AAO	Symptoms at onset	Positive sensory symptoms	Motor UL/LL	Sensory	Ulcers	Amputations	Reflexes	CMTNS2 (CMTES2)
Family A											
III-2	54	M	Mid 30s	Reduced sensation in feet	Shooting pains	N/N	Pin to mid shin	No	No	Preserved	(6/28)
III-8	65	F	Early 40s	Numbness in feet	Shooting pains	N/mild distal weakness	Vib to ankle, Pin to wrist and above knee	Yes	BKA, toes	Absent at ankle	13/36
III-9	71	M	40s	Numbness in feet	Shooting pains	Mild proximal and severe distal UL and LL weakness	Vib to costal margin, pin to face	Yes	No	Absent in LL	35/36
Family B											
II-1	47	M	30s	Pain in feet	Shooting pains	Mild distal UL weakness/N	Vib to ankles, pin to elbows and above knee	Yes	Toes	Absent at ankles	19/36
II-2	45	M	33	Numb feet	Shooting pains	N/N	Vib to ankles, pin to mid-palm and below knees	Yes	No	Preserved	ND

*AAO* age at onset, *UL* upper limbs, *LL* lower limbs, *CMTNS2* Charcot-Marie-Tooth Neuropathy Score version 2^20^, *M* male, *F* female, *N* normal, *Vib* vibration, *BKA* below knee amputation

**Table 2:** Neurophysiology of *SPTLC2–*S384 patients

Patient	Median				Ulnar				Radial	Common peroneal			Posterior tibial			Superficial peroneal	Sural
	DML (ms)	CV (m/s)	CMAP (mV)	SAP (μV)	DML (ms)	CV (m/s)	CMAP (mV)	SAP (μV)	SAP (μV)	DML (ms)	CV (m/s)	CMAP (mV)	DML (ms)	CV (m/s)	CMAP (mV)	SAP (μV)	SAP (μV)
A II-6	5.3	42	3.6	NR	6	28	0.6	NR									
A III-2																	
A III-8	3.0	58	7.0	NR	2.9		8.7	NR	13	NR						NR	NR
A III-9	NR			NR	NR			NR	NR	NR							
B II-1	3.9	49	6.1	NR	2.5	46	2.5	NR	2	3.5	42	3.3					
B II-2	3.7	50	8.6	7	3.2	56	9.4	2	21	NR			NR			NR	NR

*DML* distal motor latency, *CV* conduction velocity, *CMAP* compound motor action potential, *SAP* sensory action potential, *ms* milliseconds, *m/s* meters per second, *mV* millivolts, *μV* microvolts, *NR* no response

### Plasma 1-DeoxySL Are Elevated in S384F Mutation Carriers

**Fig. 2:**
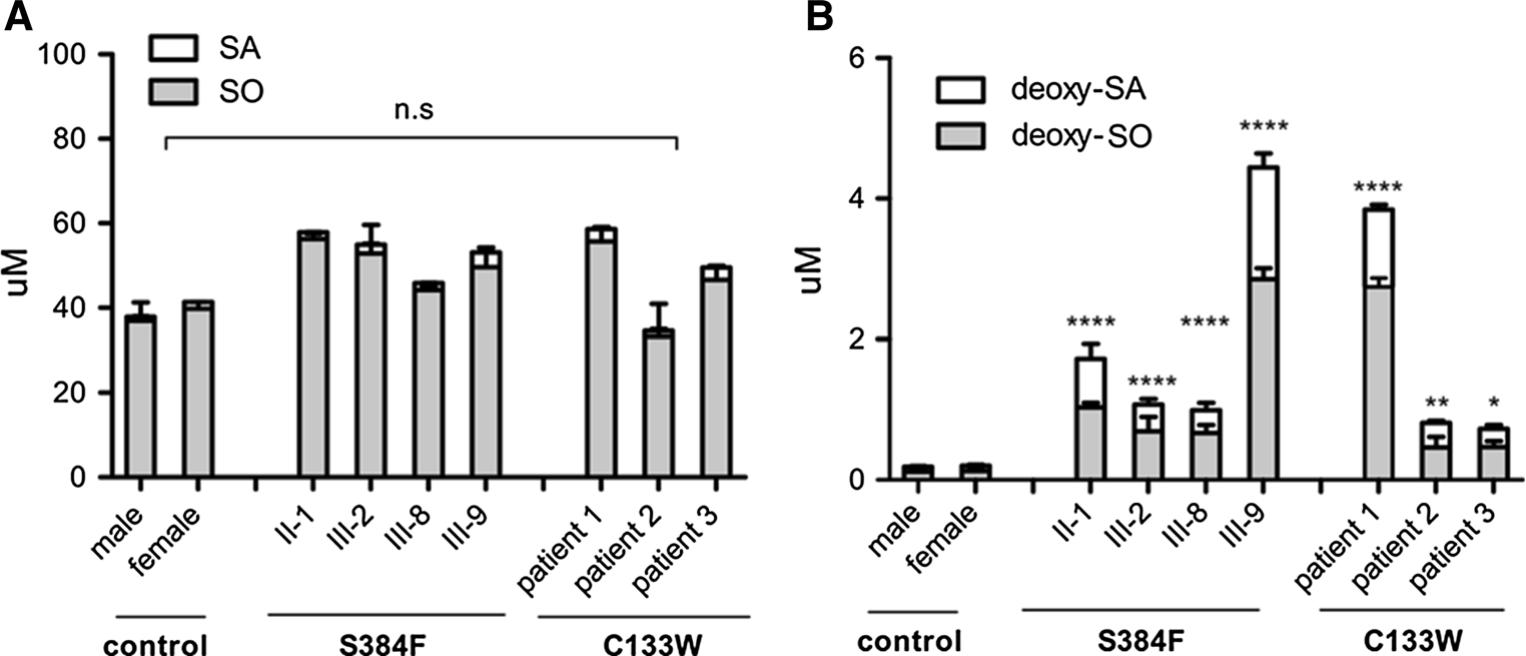
Total sphingolipids (**a**) and 1-deoxySL (**b**) in patients and controls. Total sphingolipids from plasma of two healthy controls, four *SPTLC2* S384F and three *SPTLC1*–C133W patients were extracted and analyzed by LC–MS after acid–base hydrolysis. Data are shown as mean, with *error bars* representing standard deviations. Total sphingosine (SO) levels were not different between S384F and C133W carriers compared to healthy controls (**a**), whereas 1-deoxySL (deoxy-SO, deoxy-SA) were significantly elevated in the plasma of patients with the S384F and the C133 W mutations (**b**). The total 1-deoxySL levels in the plasma of S384F and C133W carriers were comparable (**** *p* < 0.0001, *** *p* < 0.001, ** *p* < 0.01, * *p* < 0.05)

Plasma sphingolipids were analyzed in four S384F patients and compared to three HSAN1 patients with a known *SPTLC1*–C133W mutation and two healthy controls. The S384F carriers showed significantly elevated plasma 1-deoxySL levels. Typical sphingolipid levels were not altered (Fig. 2).

### SPTLC2 Is Phosphorylated In Vivo

**Fig. 3:**
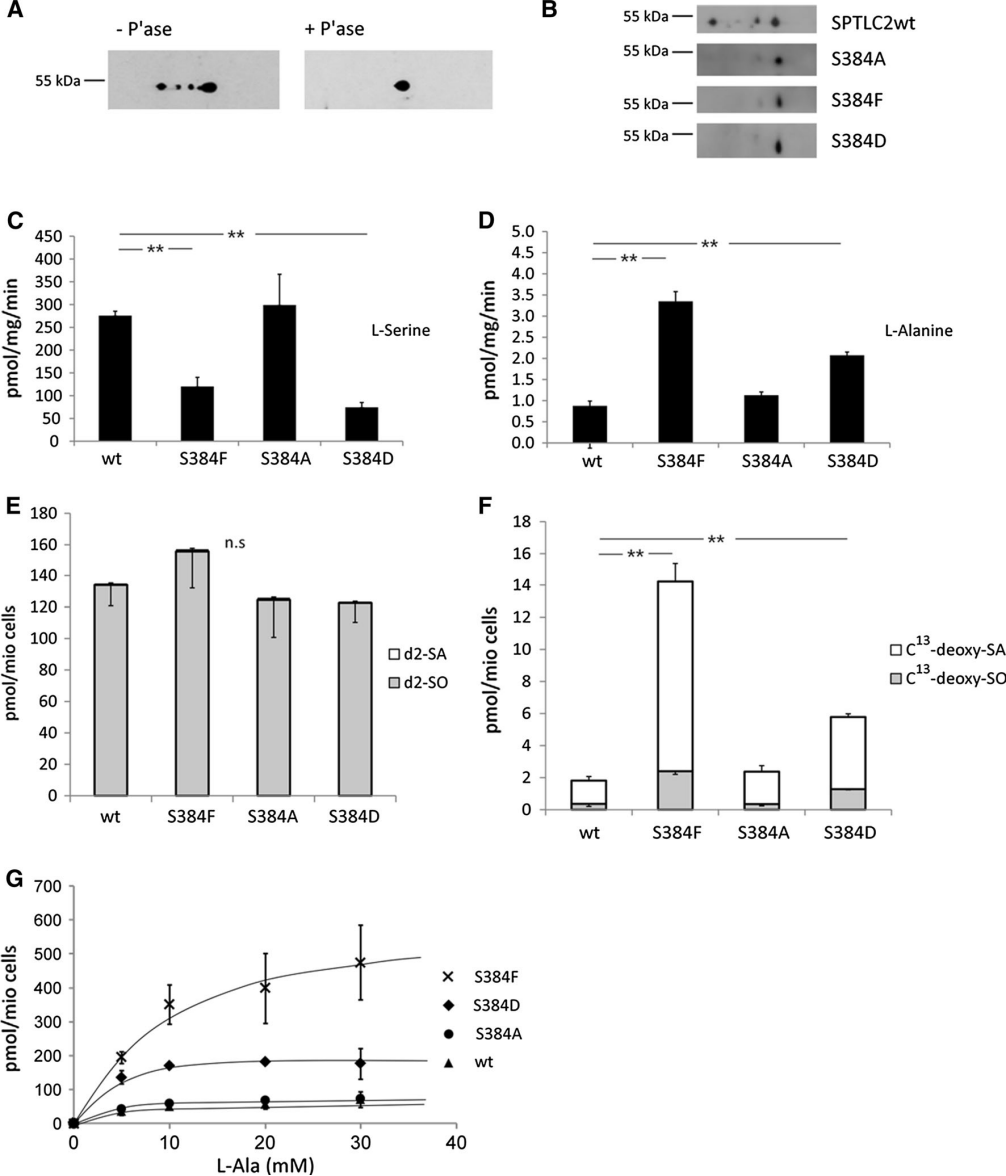
**a** Changes in the SPTLC2 phosphorylation pattern after phosphatase treatment. Total protein extract of CHO cells were extracted in the presence of phosphatase inhibitors (−P’ase) or alkaline phosphatase (+P’ase). The extract was subjected to isoelectric focusing in the first dimension and separated on a 12 % SDS-PAGE in the second dimension. SPTLC2 was detected with a polyclonal anti-SPTLC2 antibody. Phosphatase treatment resulted a single spot with a uniform isoelectric point (pI). **b** Extracts from HEK293 cells expressing SPTLC2wt, S384A, S384F or S384D were analyzed by 2D-PAGE. SPTLC2wt and mutants were detected using an anti V5-tag antibody. Isoelectric focusing showed a reduced heterogeneity in the pI for the mutants in comparison with wild type. In vitro SPT activity was analyzed in cell extract of SPTLC2wt, S384F, S384D or S384A expressing HEK293 cells either in the presence of l-serine (**c**) or l-alanine (**d**). Cells were grown in the presence of isotope-labeled d3-l-serine (1 mM) and (C_13_)-labeled l-alanine (5 mM) for 48 h. De novo formed d2-sphinganine (d2-SA) and d2-sphingosine (d2-SO) (**e**) and de novo formed C13 -deoxy-sphinganine (C13-deoxySA) and C13 -deoxy-sphingosine (C13-deoxySO) (**f**) (*Note* during the SPT reaction, one of the deuteriums of the d3-l-serine is replaced by an unlabeled hydrogen which results in the formation of d2 labeled sphinganine). Data are shown as mean with standard deviations (*N* = 3, ** *p* < 0.01). **g** 1-deoxySL formation in response to increasing amounts of l-alanine in the cell medium. SPTLC2wt and S384F, S384D and S384A expressing cells were cultured in the presence of isotope-labeled l-alanine (5–30 mM) for 48 h. The graph shows the total de novo formed 1-deoxySLs as a sum of isotope-labeled deoxy-SA and deoxy-SO

A previous phospho-proteomic screen revealed two residues in SPTLC2 (S384 and Y387) as putative phosphorylation sites (Olsen et al. 2006). Posttranslational modification of wild-type SPTLC2 was confirmed by 2D-PAGE (Fig. 3a). The heterogeneity in the isoelectric focusing (IEF) pattern was reduced to a single spot upon treatment with alkaline phosphatase. To investigate the effect of a phosphorylation at S384, we created a set of positive and negative phosphorylation mutants (Supplementary Figure 2). The mutant SPTLC2 variants were stably expressed in HEK293 cells. Expression levels for protein and mRNA were similar (Supplementary Figure 2A and B). Compared to SPTLC2wt, the positive (S384D) and the negative (S384A) mutants showed a reduced heterogeneity in the isoelectric point (pI) on 2D-PAGE (Fig. 3b), suggesting that the SPTLC2 protein is posttranslational modified at S384.

### Modifications at S384 Induce a Shift in Substrate Affinity of SPT

The activity of the mutants was analyzed in two assays. At cell-free conditions, we observed a decreased canonical SPT activity with l-serine and an increased activity with l-alanine for the HSAN1-associated S384F and phosphorylation-mimicking S384D mutant. This effect was not observed in the SPTLC2wt or the phosphorylation negative S384A mutant (Fig. 3c, d). These results were verified in cell culture by stable isotope labeling. The addition of isotope-labeled d3-serine and C^13^-alanine to mutant expressing HEK293 cells resulted in the time-dependent incorporation of the isotope label into de novo formed sphingoid bases. The incorporation of C^13^-alanine was significantly increased in the S384F and S384D expressing cells but not altered in SPTLC2wt or S384A expressing cells (Fig. 3e, f). In both assays, the activity with alanine was higher for the S384F mutant than for S384D. However, in contrast to the cell-free assay, we did not observe a reduced incorporation of d3-serine in the S384F and S384D expressing cells (SPT activities for the other variants are shown in Supplementary Figure 2C and D). Elevated concentrations of l-alanine in the cell culture medium increased 1-deoxySL formation in S384D but not in SPTLC2wt expressing cells (Fig. 3g).

## Discussion

HSAN1-associated mutations in SPT induce the pathological formation of an atypical class of neurotoxic 1-deoxySL. Elevated 1-deoxySL levels are consistently found in plasma of HSAN1 patients and appear to be a hallmark for the disease (Murphy et al. 2013; Penno et al. 2010; Rotthier et al. 2010). In plasma of healthy individuals, 1-deoxySL levels are typically low but found to be significantly elevated in patients with the metabolic syndrome and diabetes type 2 (Othman et al. 2012; Bertea et al. 2010). This indicates that wild-type SPT forms 1-deoxySL under conditions of impaired glucose or lipid homeostasis. As 1-deoxySL are neurotoxic, they might also contribute to the pathology of diabetes related sequelae—in particular the diabetic polyneuropathy (Othman et al. 2014). However, increasing the availability of l-alanine did not automatically increase 1-deoxySL formation by the wt SPT (Fig. 3g). This shows that 1-deoxySL are not simply formed as by-products of the normal SPT reaction indicating that additional factors are required to induce the substrate shift in the wild-type SPT. Interestingly, an unbiased systematic approach to characterize the in vivo phospho-proteome in EGF stimulated Hela cells revealed two phosphorylation sites (S384 and Y387) in SPTLC2 (Olsen et al. 2006). The functional consequence of these modifications was not investigated by the authors, but phosphorylation was shown to be independent of the EGF stimulus and did not follow a kinetic pattern (Olsen et al. 2006). Isoelectric focusing revealed heterogeneity in the isoelectric point (pI) for the SPTLC2 subunit (Fig. 3a). This heterogeneity was lost after alkaline phosphatase treatment which supports the concept that SPTLC2 is phosphorylated.

In parallel and independently, we identified a serine to phenylalanine exchange at the very same position (S384F) in two unrelated HSAN1 families. All S384F carriers were affected and showed increased plasma 1-deoxySL levels. The plasma levels of the canonical sphingoid bases (SA, SO) were not altered. Clinically, the S384F mutation was associated with a typical although late-onset HSAN1 phenotype with sensory, and to a lesser extent motor, involvement. Comparable to other HSAN1 mutations, there is a considerable clinical heterogeneity between patients even within the same family (Houlden et al. 2006; Murphy et al. 2013). The S384F mutant showed reduced activity with l-serine and increased activity with l-alanine in the cell-free activity assay (Fig. 3c). As the S384F mutation is located at a putative phosphorylation site in SPTLC2, we hypothesized that a phosphorylation at this position might also influence the substrate specificity of the enzyme. We therefore created a set of mutants which mimicked either a constitutively phosphorylated (S384D) or non-phosphorylated (S384A) state. Isoelectric focusing revealed a reduced heterogeneity in the pI for all S384 mutants strongly indicating the loss of a phosphorylation site. Both the S384F also the S384D mutants showed increased activity with l-alanine and a reduced canonical activity with l-serine in the cell-free activity assay which was not seen for the S384A mutant or the wild-type SPTLC2. Independently, we also tested the activity of other HSAN1 mutants, including the most frequently found SPTLC1–C133W mutant in our cell-free conditions (data not shown). Like for the S384F variant, we observed a reduced activity with l-serine for the C133W variant but could not see a significant incorporation of alanine under these conditions (data not shown). This contrasts other reports (Gable et al. 2010) which showed an activity with l-alanine also for the C133W variant under cell-free conditions. However, this discrepancy is likely related to differences in the experimental setup as Gable et al. analyzed the activity in microsomal preparations from yeast cells which were transfected with the human SPTLC1–C133W along with the human SPTLC2 and the human small SPT subunit a (ssSPTa). The same group showed previously that the co-expression of the small SPT subunits ssSPTa and ssSPTb resulted in a highly increased SPT activity (Han et al. 2009) which explains why the authors could also detect an activity with alanine for the SPTLC1–C133W mutant. Without the co-expression of the ssSPTa and b subunits we could typically detect an activity with l-alanine for the SPTLC2 mutants (e.g., S384F, A182P) (Murphy et al. 2013) but not for the SPTLC1 variants (data not shown).

To confirm the results from the cell-free assay, we also analyzed the mutant expressing cells in a metabolic labeling assay by adding stable isotope-labeled d3-serine and C^13^-alanine to the medium. Like for the cell-free assay, we observed a significantly increased 1-deoxySL formation in cells expressing the S384F and S384D mutant but not in cells expressing the SPTLC2wt or the S384A mutant (Fig. 3f, g). However, in contrast to the cell-free assay, we did not see a reduced incorporation of d3-serine for the mutants. This discrepancy might be explained by functional differences between the two assays. It was shown earlier that cellular SPT activity is tightly regulated by a metabolic feedback mechanism to prevent a potentially harmful overproduction of ceramides by the de novo pathway (Breslow and Weissman 2010). This regulation is mediated by a set of small proteins (ORM1 and 2 in yeast and ORMDL1, 2 and 3 in mammalian cells) which act, depending on their phosphorylation state, as reversible inhibitors for SPT (Breslow et al. 2010; Siow and Wattenberg 2012; Han et al. 2009; Han et al. 2010; Harmon et al. 2013). Like SPT, ORM proteins are integral components of the endoplasmic reticulum (ER) and it is therefore likely that such a metabolic feedback control requires an intact ER structure which is not given for the cell-free assay conditions. Therefore, the cell-free assay reflects the maximal SPT activity under saturating substrate conditions (*v*_max_), whereas the metabolic labeling assay measures physiological SPT activity in the cellular context. Interestingly, an increased activity with alanine was only seen for the S384F and S384D but not for the homologous S384A or S384E mutant (Supplementary Figure 2D). This showed that the switch between serine and alanine needs a specific structural modification in SPTLC2 and is not simply associated with an arbitrary amino acid exchange at position 384. The X-ray structure for the mammalian SPT is not available yet but the molecular structure of the prokaryotic form is solved for the two sphingolipid generating bacteria *Sphingomonas paucimobilis* and *Sphingobacterium multivorum* (Yard et al. 2007; Ikushiro et al. 2009). In contrast to the mammalian SPT, the prokaryotic SPT is a homodimer and either soluble (*S. paucimobilis*) or loosely attached to the inner cell membrane (*S. multivorum)* (Ikushiro et al. 2007). The PLP-binding motif is organized as a coil surrounded by two beta sheets (Supplementary Figure 3A). Modeling the mammalian residues S384 and Y387 into this structure indicates that Ser384 is positioned directly in the core of the PLP-binding motif, pointing outside toward the PLP-binding pocket, whereas Y387 is rather localized on the beta sheet pointing away from the binding pocket (Supplementary Figure 3B). This indicates that S384 rather than Y387 would be accessible for a kinase from outside. The observation that a specific phosphorylation of SPT acts as a molecular switch to shift the substrate specificity of the enzyme from serine to alanine supports a physiological role for the 1-deoxySLs in the cellular lipid metabolism.

In summary, we showed that S384F is a novel mutation in HSAN1 and that the substrate specificity of the wild-type SPT might be dynamically regulated by a phosphorylation at this position. However, the physiological consequences, underlying regulatory mechanisms and associated signaling pathways need to be investigated in further studies.

## Electronic supplementary material

Below is the link to the electronic supplementary material.

Supplementary material 1 (DOCX 933 kb)
