# Supplementary material for: Novel HSAN1 Mutation in Serine Palmitoyltransferase Resides at a Putative Phosphorylation Site That Is Involved in Regulating Substrate Specificity
Source: Neuromolecular Med. 2015 Jan 8;17(1):47–57. doi: 10.1007/s12017-014-8339-1 (PMC4326654; doi:10.1007/s12017-014-8339-1)
Supplement: Supplementary file 1 — Supplementary material 1 (DOCX 933 kb) [file 12017_2014_8339_MOESM1_ESM.docx]

**Supplementary Material**


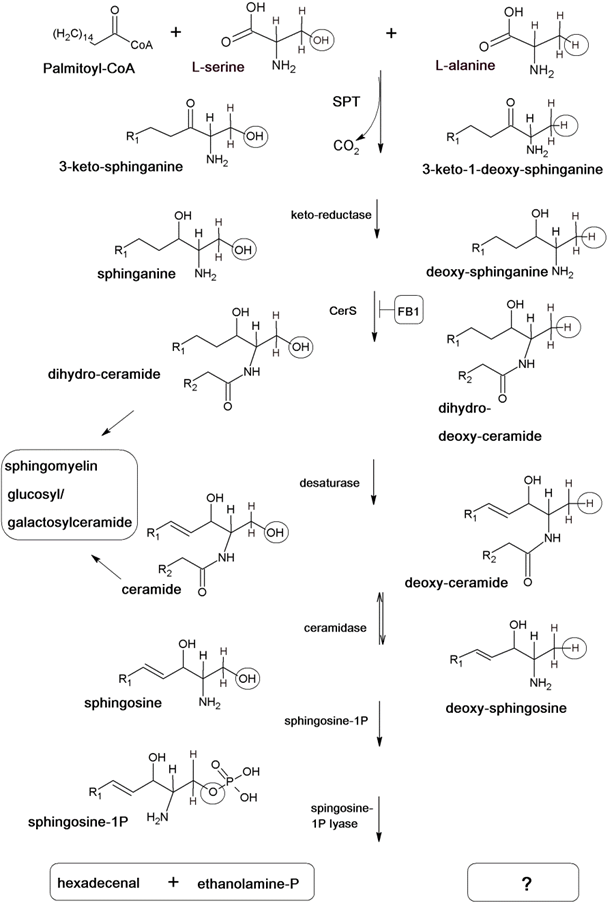


**SUPPLEMENTARY FIGURE 1:** *De novo* ceramide synthesis is initiated by the conjugation of palmitoyl-CoA with L-serine to form 3-keto-sphinganine which is subsequently reduced to sphinganine (SA). SA is acylated by ceramide synthase (CerS) and desaturated by ceramide desaturase (DES) to form ceramide. The degradation starts with the de-acetylation of ceramide by ceramidase forming sphingosine (SO) which is phosphorylated by SO-Kinase and finally degraded to hexadecenal and phosphoethanolamine by sphingosine-1-phospate lyase (SO1P-lyase). The use of L-alanine results in the formation of deoxy-sphinganine that is acylated to deoxy-dihydro-ceramide and deoxy-ceramide. The missing C1-hydoxyl-group prevents the conversion of deoxy-ceramides into complex sphingolipids, like sphingomyelins or glycosphingolipids but also the formation of sphingosine-1-phophate as a catabolic intermediate.

**
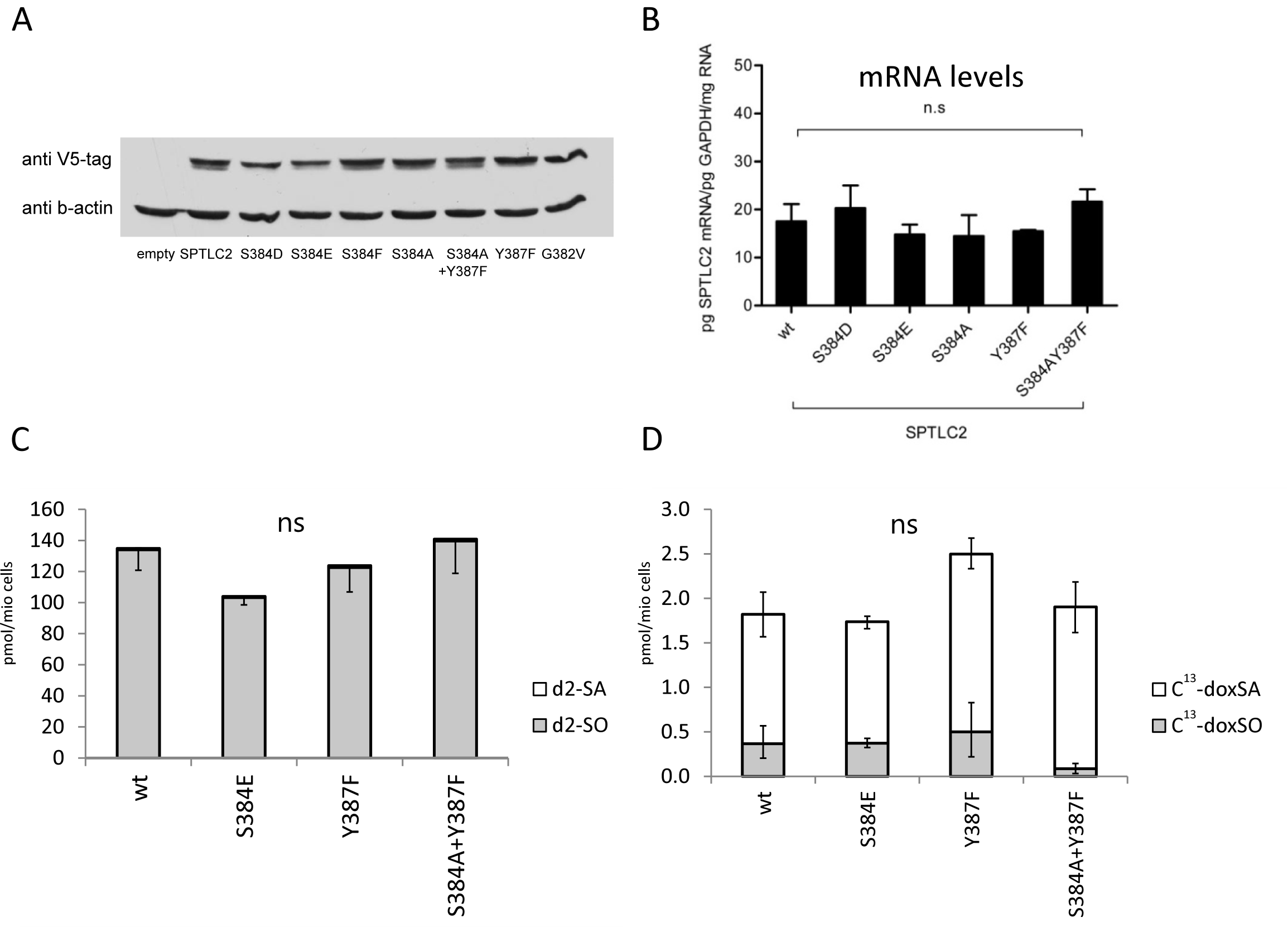
**

**SUPPLEMENTARY FIGURE 2: (A)** Expression levels of wild type SPTLC2 and the SPTLC2 mutants in stably transfected HEK293 cells. Proteins were separated on a 12% SDS Page and detected by a V5-tag antibody. Beta-actin was used as the loading control. (**B)** mRNA levels of wild type SPTLC2 and mutants in stably transfected HEK293 cells. GAPDH was used for normalization.

Incorporation of **(C)** isotope labeled (2,3,3)-d3 L-serine (1mM) and **(D)** (C13)- L-alanine (5mM) in HEK293 cells expressing SPTLC2wt or the SPTLC2 mutants S384E, Y384F or the double negative mutant S384A+Y387A. Cells were incubated for 24h with the isotope labelled amino acids. After harvesting the total lipids were extracted, hydrolyzed and the isotope labeled sphingoid bases analyzed by LC-MS. We observed a slight reduction in the canonical activity for the S384E mutant and a slightly increased 1-deoxySL formation for the Y387F mutant. The double mutant (S384A+Y387A) was comparable to the SPTLC2wt. All data are shown as mean, with error bars representing standard deviations (N=3, *p < 0.05).


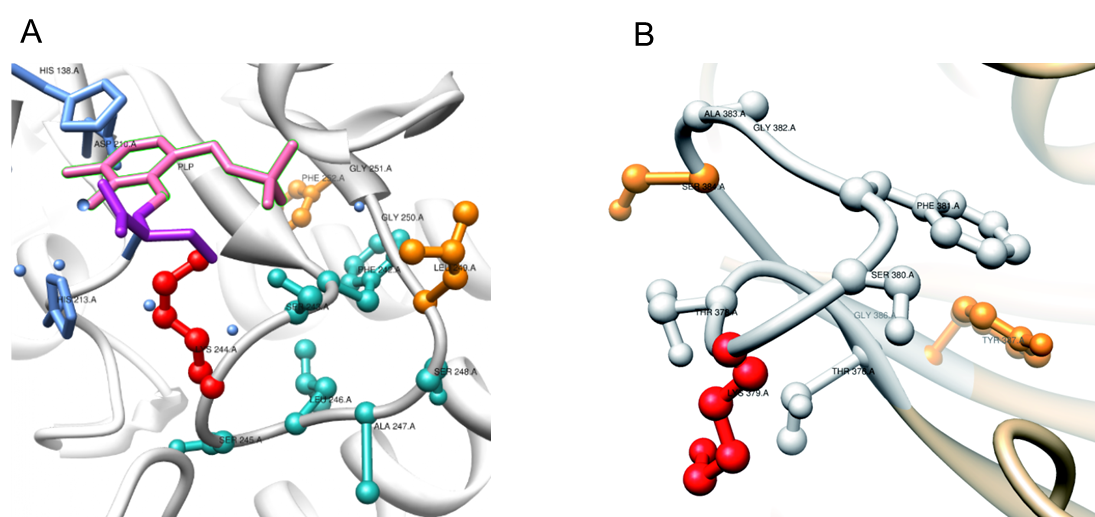


**SUPPLEMENTARY FIGURE 3:** The mammalian SPT is presumably an octamer with a mass of 460kD composed of four heterodimers each formed by one SPTLC1 and either a SPTLC2 or SPTLC3 subunit. The x-ray structure for mammalian SPT is not resolved yet but the molecular structure of the prokaryotic forms is resolved from the two sphingolipid generating bacteria *Sphingomonas paucimobilis (*[*Yard et al. 2007*](#_ENREF_3)*)* and *Sphingobacterium multivorum* ([Ikushiro et al. 2009](#_ENREF_1)). Prokaryotic SPT is a homodimer and either soluble (*S. paucimobilis*) or loosely attached to the inner cell membrane (*S.multivorum) (*[*Ikushiro et al. 2007*](#_ENREF_2)*)*. The PLP binding motif in the x-ray structure from *S.multivorum* is organized as a coil surrounded by two beta-sheets.

**(A)** Structure of SPT in *sphingobacterium multivorum* (PDB:3A2B) was illustrated using UCSF Chimera 1.6.2. Leu249 (=S384 in humans) is located in a coil structure surrounded by 2 beta-sheets whereas Phe252 (=Y387 in humans) is found on the beta-sheet outside the PLP-binding motif sequence. Other aminoacids located on the coil structure are illustrated in turquoise (Ser248, Ala247, Leu246, Ser245, Ser243, Phe242). The substrate (serine-PLP in purple-pink) is bound to Lys244 (purple-pink). His138 and His213 (blue) are important for coordinating the right position of the substrate. Leu249 and Phe252 are illustrated in orange.

**(B)** Modeling of human SPTLC2 phosphosites in *sphingobacterium multivorum*

The sequence of the mammalian SPTLC2 subunit was modeled into the protein structure of *S. multivorum* (PDB:3A2B) using SWISS-MODEL (http://swissmodel.expasy.org). Both phosphosites (Ser384 and Tyr387) are shown in orange. The Schiff-base forming Lys379 and the Ser384 are both located on the coil structure of the inner core of the PLP-binding motif (FT**K**SXXX**X_Ser_**G) whereas the second Tyr387 is located on the beta-sheet.

Ikushiro, H., Islam, M. M., Okamoto, A., Hoseki, J., Murakawa, T., Fujii, S., et al. (2009). Structural insights into the enzymatic mechanism of serine palmitoyltransferase from Sphingobacterium multivorum. *J Biochem, 146*(4), 549-562, doi:10.1093/jb/mvp100.

Ikushiro, H., Islam, M. M., Tojo, H., & Hayashi, H. (2007). Molecular characterization of membrane-associated soluble serine palmitoyltransferases from Sphingobacterium multivorum and Bdellovibrio stolpii. *J Bacteriol, 189*(15), 5749-5761.

Yard, B. A., Carter, L. G., Johnson, K. A., Overton, I. M., Dorward, M., Liu, H., et al. (2007). The structure of serine palmitoyltransferase; gateway to sphingolipid biosynthesis. *J Mol Biol, 370*(5), 870-886.
